# Prediction of Severe Retinopathy of Prematurity Using the Winrop Algorithm in a Cohort from Malopolska. a Retrospective, Single-center Study

**DOI:** 10.34763/devperiodmed.20172104.336343

**Published:** 2018-01-02

**Authors:** Mateusz Jagła, Anna Peterko, Katarzyna Olesińska, Izabela Szymońska, Przemko Kwinta

**Affiliations:** 1 Chair of Pediatrics, Jagiellonian University, Collegium Medicum, Cracow, Poland; 2 Student Research Group, Chair of Pediatrics, Jagiellonian University, Collegium Medicum, Cracow, Poland

**Keywords:** retinopathy of prematurity, WINROP algorithm, prematurity, retinopatia wcześniaków, algorytm WINROP, wcześniactwo

## Abstract

**Introduction:**

Retinopathy of prematurity (ROP) is one of the leading avoidable causes of blindness in childhood in developed countries. Accurate diagnosis and treatment are essential for preventing the loss of vision. WINROP (https://www.winrop.com) is an online monitoring system which predicts the risk for ROP requiring treatment based on gestational age, birth weight, and body weight gain.

**Aim:**

To validate diagnostic accuracy of the WINROP algorithm for the detection of severe ROP in a single centre cohort of Polish, high-risk preterm infant population.

**Material and methods:**

Medical records of neonates born before 32 weeks of gestation admitted to the third level neonatal centre in a 2-year retrospective investigation 79 patients were included in the study: their gestational age, birth weight and body weight gain were set in the WINROP system. The algorithm evaluated the risk for ROP divided into low or high-risk of disease and identified infants with high risk of developing severe ROP (type 1 ROP).

**Results:**

Out of 79 patients 37 received a high-risk alarm, of whom 22 developed severe ROP. Low-risk alarm was triggered in 42 infants; five of them developed type 1 ROP. The sensitivity of the WINROP was found to be 81.5% (95% CI 61.9-93.7), specificity 71.2% (95% CI 56.9-82.9), negative predictive value (NPV) 88.1% (95% CI 76.7-94.3), and positive predictive value (PPV) 59.5 (95% CI 48.1-69.9), respectively. The accuracy of the test significantly increased after combined WINROP and surfactant therapy as an additional factor - sensitivity 96.3% (95% CI 81.0-99.9), specificity 63.5% (95% CI 49.0-76.4), NPV 97.1% (95% CI 82.3-99.6), and PPV 57.8 (95% CI 48.7-66.4).

**Conclusions:**

The WINROP algorithm sensitivity from the Polish cohort was not as high as that reported in developed countries. However, combined with additional factors (e.g. surfactant treatment) it can be useful for identifying the risk groups of sight-threatening ROP. The accuracy of the WINROP algorithm should be validated in a large multi-center prospective study in a Polish population of preterm infants.

## Streszczenie

**Wstęp:**

Retinopatia wcześniaków (ROP) jest najczęstszą z możliwych do uniknięcia przyczyn ślepoty u dzieci w krajach rozwiniętych. Jej właściwe rozpoznanie i leczenie są niezbędne do zachowania wzroku. WINROP (https://www.winrop.com) jest systemem komputerowym, przewidującym ryzyko wystąpienia ROP wymagającego leczenia, w oparciu o wiek ciążowy, urodzeniową masę ciała oraz jej przyrost.

**Cel:**

Sprawdzenie wartości diagnostycznej algorytmu WINROP w wykrywaniu ciężkiego ROP w grupie wcześniaków wysokiego ryzyka.

**Materiał i metody:**

Analizie retrospektywnej poddano dokumentację medyczną 79 noworodków urodzonych przed 32 tygodniem trwania ciąży, przyjętych do III-rzędowego oddziału noworodkowego przez okres 2 lat. Wiek ciążowy, masę urodzeniową oraz przyrost masy ciała wprowadzano do systemu WINROP. Algorytm oceniał ryzyko rozwoju ROP na niskie bądź wysokie i wskazywał dzieci narażone na ryzyko rozwoju ciężkiej postaci ROP (ROP typu 1).

**Wyniki:**

Z 79 pacjentów objętych badaniami alarm wysokiego ryzyka otrzymało 37 pacjentów, z których 22 rozwinęło ROP typu 1. 42 noworodków otrzymało alarm niskiego ryzyka, u 5 z nich stwierdzono ROP typu 1. Czułość WINROP została oceniona na 81,5% (95% CI 61,9-93,7), specyficzność 71,2% (95% CI 56,982,9), wartość predykcyjna ujemna (NPV) 88,1% (95% CI 76,7-94,3), wartość predykcyjna dodatnia (PPV) 59,5 (95% CI 48,1-69,9). Dokładność znacznie wzrosła po dołączeniu do WINROP terapii surfaktantem jako dodatkowego czynnika - czułość 96,3% (95% CI 81,0-99,9), swoistość 63,5% (95% CI 49,0-76,4), NPV 97,1% (95% CI 82,3-99,6), i PPV 57,8 (95% CI 48,7-66,4).

**Wnioski:**

Czułość WINROP w kohorcie badanych noworodków była niższa w porównaniu z wynikami autorów w krajach wysokorozwiniętych. Jednak w połączeniu z dodatkowym czynnikiem ryzyka (np. terapią surfaktantem) może być użyteczną metodą identyfikacji grupy ryzyka rozwoju ciężkiego ROP. Dokładność algorytmu WINROP powinna zostać potwierdzona w dużym, wieloośrodkowym, prospektywnym badaniu polskiej populacji noworodków urodzonych przedwcześnie.

## Introduction

Retinopathy of prematurity (ROP) is a developmental vascular proliferative disease. It is a serious problem among the preterm infant population. The frequency of ROP is estimated between 10% to 21% among children under 1,250 g, however, it can reach even up to 68% [1, 2, 3]. Retinopathy of prematurity should be effectively and quickly detected, because if left untreated, it can lead to visual impairment or even vision loss [4]. The early detection of problems provides an opportunity to apply proper prevention. The effective screening programme should identify infants with ROP that require treatment. Currently the gold standard test for the diagnosis of ROP is ophthalmological examination, but it is a stressful and painful procedure [5]. In contrast to that, the WINROP algorithm is a non-invasive, simple and inexpensive method of predicting ROP risk.

The online WINROP (weight, insulin-like growth factor [IGF], neonatal, retinopathy of prematurity [ROP]) algorithm (https://www.winrop.com) can be used to identify infants with increased risk for developing severe ROP [4]. It was developed in Gothenburg, Sweden based on their studies of IGF-1 showing a correlation between a prolonged period of low-level serum IGF-1 and ROP [6, 7]. WINROP calculated on the basis of birth weight, postnatal weight gain, and gestational age determines the approximate IGF-1 levels and in this way identifies the risk of developing sight-threatening ROP [8].

The WINROP surveillance system has been validated in several studies. In highly developed countries (Sweden and United States) the accuracy of the test was higher, as opposed to less and moderately developed countries (Taiwan, Turkey, Korea) [8, 9, 10]. ROP newborns in those countries were more mature and the WINROP algorithm omitted more infants with severe ROP.

The vascular endothelial growth factor (VEGF) and insulin-like growth factor 1 are human proteins, which are necessary for the normal development of retinal blood vessels. Prolonged low levels of serum IGF-1, which usually affect preterm infants, may finally lead to the development of ROP [6]. Gestational age and birth weight cannot be considered in isolation from other risk factors of ROP [8]. Retinopathy of prematurity is also associated with other risk factors, such as assisted ventilation for longer than one week, surfactant therapy, cumulative illness severity, low caloric intake, hyperglycemia and insulin therapy, sepsis, fluctuations in blood gas measurements, intraventricular hemorrhage (IVH), bronchopulmonary dysplasia (BPD) and early administration of erythropoietin for the treatment of anemia of prematurity [11, 12, 13, 14, 15, 16, 17, 18].

## Aim of the study

The aim of this study was to validate the diagnostic accuracy of the WINROP algorithm for detection of severe ROP in a single-centre cohort of preterm infants from Malopolska, Poland.

## Material and methods

### Patients

The infants were eligible for the study if they were born below 32 weeks’ gestation and were admitted to the Neonatal Intensive Care Unit (NICU) of the Department of Pediatrics, Jagiellonian University, before the second week of life between 2013 and 2015. The vast majority of the newborns were admitted within 24 hours after birth. Gestational age was determined based on fetal biometry measures taken in the first trimester of pregnancy. The New Ballard Score maturational assessment of gestational age was additionally performed if there were any doubts (birth weight for gestational age below 10 percentile or above 90 percentile). The NICU is a level III centre located in Malopolska, the southeastern region of Poland. There is no maternity department in the centre, all the patients are transported from referring hospitals, frequently with surgical conditions, multiple birth defects or severe infections. The patient’s detailed data were prospectively recorded in a computer database daily. The body weight of the infants was measured daily until discharge.

### Retinopathy of prematurity examination and treatment

All the infants were examined according to the diagnostic guidelines of The International Classification of Retinopathy of Prematurity revisited [19]. Ophthalmologic examination in Poland was recommended for all infants with a birth weight less than 1500 g or gestational age below 32 weeks and infants with birth weight between 1500 and 2000 g, or gestational age over 32 weeks with an unstable clinical course [20]. The frequency of retinal examinations increased after detecting ROP signs. In our study the examination was performed by a pediatric ophthalmologist after mydriasis with tropicamide and phenylephrine using a binocular indirect ophthalmoscope (Keeler Instruments Inc., US). The stages of retinopathy of prematurity were determined according to the International Classification of ROP (1 to 5 stages). The infants underwent therapy when they developed type 1 ROP. Type 1 ROP is defined as any of the following: any stage of ROP with plus disease in zone I, stage 3 ROP without plus disease in zone I or stage 2 or 3 ROP with plus disease in zone II.

Treatment was performed for infants with indications characterized in Early Treatment for Retinopathy of Prematurity (ETROP) criteria [21]. Laser photocoagulation treatment was performed by infrared laser photocoagulator (OcuLight® SL Iridex Corporation, US).

### WINROP screening

The WINROP algorithm predicts the risk of proliferative ROP and divides infants into two groups with low and high risk of developing sight threatening retinopathy. The algorithm is based on three parameters: gestational age, birth weight, and weight recorded weekly until the alarm or 36 weeks’ postmenstrual age (PMA). The results of WINROP screening were compared with ophthalmologic examination.

### Statistical analysis

Continuous variables were presented as mean (±SD) or median (±IQR), qualitative variables as numbers and percentages (n, %). Differences between the groups were compared using Fisher exact test and Mann-Whitney U test. The diagnostic power of a WINROP algorithm in the diagnosis of ROP was determined by calculating sensitivity, specificity, positive predictive value (PPV), and negative predictive value (NPV) with 95% confidence intervals. The results of ophthalmologic examination were adopted as the “gold standard”. In addition, the level of accuracy between WINROP algorithm results and the final diagnosis was calculated using Cohen’s kappa coefficient κ. In all the analyses the differences were defined as significant when the p-value was less than 0.05. Statistical analyses were performed using the MedCalc statistical package, ver. 17.2 (MedCalc Software, Ostend, Belgium).

## Results

### ROP outcome

Overall 79 preterm infants were initially screened. Eight infants were excluded, because they were born before 23 weeks of gestational age (n=1), died before the 28^th^ day of life (n=6) or had incomplete body weight data (n=1). Finally, 79 patients were eligible for further analysis.

Patients were divided into 2 groups: Non-type 1 ROP and type 1 ROP. The non-type 1 ROP group included patients without ROP and children with ROP, but in a low stage of disease without indications for laser retinal photocoagulation. The clinical and demographic characteristics of the study groups are presented in table I.

**Table I:** Characteristics of the participating preterm infants. Tabela I. Charakterystyka grupy wcześniaków włączonych do badania.

Characteristic *Charakterystyka*	All *Wszyscy* n = 79	Non-type 1 ROP *ROP typu innego niż 1* n=52	Type 1 ROP *ROP typu 1* n=27	p value *wartość p*
Gender, male, n (%) *Płeć męska, n (%)*	49 (62)	31 (60)	18 (67)	0.3591
Gestational age, weeks, median (IQR) *Wiek* *ciążowy*, *tygodnie*, *mediana* *(IQR)*	28 (26-30)	29 (28-30,5)	26 (25-27)	<0.001^1^
Birth weight, g, median (IQR) *Masa urodzeniowa, g, mediana (IQR)*	1095 (900-1315)	1216 (1055-1396,5)	893 (760-1000)	<0.001^1^
CRIB II score, median (IQR) *Skala CRIB II, mediana (IQR)*	7 (5-9)	6 (4-7)	9 (8-11)	0.001^1^ <
GCS prenatal, n (%) *Steroidy* *prenatalne*, *n (%)*	47 (59)	27 (52)	20 (74)	0.047^2^
Surfactant, n (%) *Surfaktant, n (%)*	44 (56)	24 (46)	20 (74)	0.0162
IVH grade III or IV, n (%) *IVH stopień III lub IV, n (%)*	9 (11)	4 (8)	5 (19)	0.1442
PDA ibu, n (%)	35 (44)	18 (35)	17 (63)	0.015^2^
PDA lig, n (%)	12 (15)	3 (6)	9 (33)	0.002^2^
Hyperglycaemia, Hiperglikemia*, n (%)	10 (13)	5 (10)	5 (19)	0.252^2^
Sepsis, n (%) *Sepsa*, *n* *(%)*	38 (48)	21 (40)	17 (63)	0.047^2^
NEC, n (%)	4 (5)	3 (6)	1 (4)	0.577^2^
BPD, n (%)	21 (27)	6 (12)	15 (56)	<0.001^2^
Birth defects, n (%) *Wady wrodzone, n (%)*	11 (14)	7 (13)	4 (15)	0.557^2^

1 Mann-Whitney U test/*Test U Manna-Whitneya* ^2^Fisher exact test/*Dokładny test Fishera* BPD − bronchopulmonary dysplasia/dysplazja oskrzelowo-płucna; CRIB II − Clinical Risk Index for Babies Scoring System II/*kliniczna skala oceny ryzyka zgonu dla noworodków*; Hyperglycemia – more than 5% of blood glucose measurements over 180 mg/dL/*hiperglikemia – więcej niż 5% pomiarów glukozy we krwi o wartości ponad 180 mg/dL*; IVH − intraventricular hemorrhage/*krwawienie dokomorowe*; NEC − necrotizing enterocolitis/*martwicze zapalenie jelit*; PDA – hemodynamically significant patent ductus arteriosus closed by ibuprofen (ibu) or by surgical ligation (lig)/*hemodynamicznie istotny przewód tętniczy zamknięty przez ibuprofen (ibu) lub przez ligację chirurgiczną*; ROP − retinopathy of prematurity/*retinopatia wcześniaków*.

### WINROP outcome

In our study, 79 infants entered into the online surveillance system obtained an algorithm score. The low-risk alarm was triggered in 42 infants (53.2%). Of the infants in the group that received a low-risk alarm, 5 developed type 1 ROP and were treated with laser retinal photocoagulation. All of these infants had such complications of prematurity as BPD (n=3), IVH (n=3), or required surfactant therapy (n=4) (tab. II).

**Table II:** Characteristics of 5 infants who developed type 1 ROP in the low-risk alarm group. Tabela II. Charakterystyka 5 noworodków które rozwinęły ROP typu 1 z grupy alarmu niskiego ryzyka.

	Gender *Płeć*	Gestational age, weeks *Wiek ciążowy, tygodnie*	Birth weight *Masa urodzeniowa (g)*	BPD *Dysplazja oskrzelowo-płucna*	Sepsis *Sepsa*	Surfactant *Surfaktant*	Hyperglycemia *Hiperglikemia*	Anaemia *Niedokrwistość*	IVH *Krwawienia dokomorowe*
1	Male *Męska*	26	1130	+	+	+	-	+	+
2	Female *Żeńska*	26	1050	-	-	+	-	-	-
3	Male *Męska*	28	1270	-	-	-	-	-	-
4	Male *Męska*	24	800	+	-	+	-	+	+
5	Male *Męska*	23	720	+	+	+	-	+	+

Abbreviations/Skrófy: BPD - bronchopulmonary *dysplasia/dysplozjo oskrzelowo-płucna;* Hyperglycaemia – more than 5% of blood glucose measurements over 180 mg/dL*/hiperglikemia – więcej niż 5% pomiarów glukozy we krwi o wartości ponad 180 mg/dL;* IVH – intraventricular hemorrhage/*krwaowienie dokomorowe*.

The high-risk alarm occurred in 37 (46.8%) infants and 22 of the group developed type 1 ROP. WINROP alerted the risk of developing ROP median 35 days (IQR 28-40) before an ophthalmologist confirmed it in the examination and a median 53.5 days (IQR 40-68) before the laser therapy.

### Test characteristics

The sensitivity, specificity, positive and negative predictive values of the WINROP algorithm in predicting type 1 ROP for the study group were calculated (tab. III). There was also the accuracy of the test calculated after combined WINROP and surfactant therapy as an additional risk factor associated with ROP (tab. IV).

**Table III:** Diagnostic performance of WINROP in predicfing the type 1 ROP. Tabela III. Diagnostyczna skuteczność WINROP w przewidywaniu ROP typu 1

	Type 1 ROP *ROP typu 1*	Non-type 1 ROP *ROP typu innego niż 1*
WINROP High-risk alert (n=37) Alarm wysokiego ryzyka (n=37)	22	15
WINROP Low-risk alert (n=42) Alarm niskiego ryzyka (n=42)	5	37
Sensitivity, % (95% CI) *Czułość, % (95% CI)*	81.48 (61.92-93.70)	
Specificity, % (95% CI) *Specyficzność, % (95% CI)*	71.15 (56.92-82.87)	
PPV, % (95% CI)	59.46 (48.08-69.91)	
NPV, % (95% CI)	88.10 (76.70-94.33)	
Cohen’s kappa coefficient *Współczynnik kappa Cohena*	0.483	
AUC, (95% CI)	0.76 (0.65 to 0.85)	

Abbreviations/Skróty: PPV − positive predictive value/*wartość predykcyjna dodatnia*; NPV − negative predictive value/*wartość predykcyjna ujemna*; AUC − area under the curve/*pole pod krzywą*

**Table IV:** Diagnostic performance of WINROP + surfactant therapy in predicting of type 1 ROP. Tabela IV. Diagnostyczna skuteczność WINROP + terapia surfaktantem w przewidywaniu ROP typu 1.

	Type 1 ROP *ROP typu 1*	Non-type 1 ROP *ROP typu innego niż 1*
WINROP High-risk alert or surfactant therapy (n=45) *Alarm wysokiego ryzykalub terapia surfaktantem (n=45)*	26	19
WINROP Low-risk alert (n=34) *Alarm niskiego ryzyka (n=34)*	1	33
Sensitivity, % (95%CI) *Czułość, % (95%CI)*	96.30 (81.03-99.91)	
Specificity, % (95%CI) *Swoistość, % (95%CI)*	63.46 (48.96-76.38)	
PPV, % (95%CI)	57.78 (48.70-66.36)	
NPV, % (95%CI)	97.06 (82.67-99.56)	
Cohen’s kappa coefficient *Współczynnik kappa Cohena*	0,51	
AUC, (95%CI)	0.80 (0.69-0.88)	

Abbreviations/Skróty: PPV − positive predictive value/*wartość predykcyjna dodatni*a; NPV − negative predictive value/*wartość predykcyjna ujemna*; AUC − area under the curve/*pole pod krzywą*

## Discussion

In our analysis the WINROP algorithm correctly identified 22 out of 27 (81.5%) preterm infants, who developed type 1 ROP and required treatment according to ETROP criteria. Five newborns who developed sightthreatening ROP were omitted by that online surveillance system (false negative results). In several studies from Sweden and the United States the sensitivity of the WINROP algorithm in detecting proliferating ROP reached 100% [22, 23, 24]. The sensitivity of WINROP in different studies is heterogeneous, with a range of 64.7% to 100% [8, 9, 10, 24]. In our cohort group the sensitivity of 81.5% is comparable to the value estimated in Turkey or Taiwan [8, 9]. There are some reasons that could have had an impact on those discrepancies between the studies.

The disparity possibly came from the fact that the WINROP algorithm was developed and validated in children from Sweden and the United States. The sensitivity validated on a population from less developed countries was significantly lower [9].

Previous study outcomes suggested that larger, more mature infants develop severe ROP in lower developed countries compared with highly developed countries [25]. In the results of those studies it was said that the mean birth weight of premature patients was lower in highly developed countries, ranging from 737 to 763g than in less developed countries, where it ranged from 903 to 1527 g. The gestational age of infants with severe ROP was also distinct: 25.3 to 25.6 weeks in highly developed countries compared with 26.3 to 33.5 weeks in less developed countries. Various mixes of cases, neonatal care, survival rates and screening practices could have impact on those conditioning. In our study the values of those parameters were heterogeneous. The gestational age of infants not identified by WINROP was 23-28 weeks and the birth weight 720-1270 grams.

The gestational age and birth weight are major risk factors significantly associated with ROP. There are also multiple contributing risk factors, including: bronchopulmonary dysplasia, intraventricular hemorrhage, patent ductus arteriosus, mechanical ventilation and sepsis. Surfactant therapy in multivariate analysis included 38 possible risk factors which were also associated with higher incidence of ROP. Surfactant therapy decreased the mortality of extremely low-birth-weight infants, i.e. patients most at risk for retinopathy of prematurity. Selecting this factor has contributed to enhancing WINROP accuracy [11, 17, 26].

Several studies from Korea and Turkey have shown that patients who were not identified by the WINROP algorithm had additional risk factors, including BPD, IVH, necrotizing enterocolitis (NEC) or sepsis, which added to the analysis and significantly improved the sensitivity of the WINROP algorithm results [8, 10]. In our study 20 out of 27 patients with type 1 ROP and 4 out of 5 patients omitted by WINROP required surfactant therapy [27]. The modified algorithm included surfactant therapy as an additional risk factor and increased sensitivity to 96.3% [26, 27].

Patients admitted to the Neonatal Intensive Care Unit of the Department of Pediatrics, Jagiellonian University, were a high-risk population with significant disorders including congenital defects, surgical disorders and severe prematurity complications. Infants with serious postnatal problems were transported from referring hospitals. These aspects may be especially relevant to confirming that the infant population is highly specific. Screening examination of premature infants for retinopathy of prematurity is regularly performed due to the significant number of hospitalized patients with these diseases. The low frequency of antenatal steroid therapy among hospitalized patients (47/79) is a remarkable risk factor of ROP occurring. Antenatal dexamethasone or betamethasone are associated with a reduced risk for ROP [28, 29].

The study was limited by a comparatively small number of patients and confirmation of the disease only by a single ophthalmologist, without inter-observer variability assessment. Diagnosis and documentation of ophthalmoscopic findings may be heavily subjective. It was documented that the agreement on plus disease diagnosis among pediatric ophthalmologists is low [30].

## Conclusions

WINROP is a useful noninvasive screening surveillance system which can predict proliferative retinopathy of prematurity for very low birth weight infants in highly developed countries. As a result of repetitive falsely negative outcomes, it demonstrates insuficient sensitivity to becoming a screening test in a Polish cohort group. Modification of the WINROP algorithm by taking different factors into account, like surfactant therapy after birth, increases the test’s sensitivity and only individual cases remain falsely negative. The accuracy of the WINROP algorithm should be validated in large multi-center prospective studies in the Polish population of preterm infants.
